# EMERITI PROFESSORS’ INTERGENERATIONAL CONNECTION: QUALITATIVE RESEARCH EXPLORING PERCEPTIONS AND SATISFACTION

**DOI:** 10.1093/geroni/igad104.0087

**Published:** 2023-12-21

**Authors:** Karen Devereaux Melillo, Ramraj Gautam, Lisa Abdallah, Patcharee Wangwun

**Affiliations:** University of Massachusetts Lowell, Lowell, Massachusetts, United States; University of Massachusetts Lowell, Lowell, Massachusetts, United States; University of Massachusetts Lowell, Lowell, Massachusetts, United States; University of Massachusetts Lowell, Lowell, Massachusetts, United States

## Abstract

UMass Lowell, designated an AFU in 2019, launched AFU Inventory and Climate Surveys in 2020; respondents identified “involving retired faculty in university activities’ (AFU Principle 9) as an area for improvement. A follow-up study, guided by the PEACE Model (Levey, 2018), was undertaken to reduce students’ negative stereotypes about older adults and promote positive experiences through active engagement with professor emeriti. One study objective was to explore the experiences of emeriti professors’ in engaging online with Intro to Gerontology students in a novel intergenerational classroom activity. Emeriti professors met twice for one hour each with a group of 4-5 students. Following the activity, emeriti professors were invited to participate in one-hour qualitative interviews. The purpose was to explore their perceptions of, and satisfaction with, the experience in having engaged online with students. Nine of the 12 emeriti professors agreed to participate. The Zoom interviews were conducted December 2022 and January 2023 by two researchers. Qualitative content analysis coding was carried out individually by four researchers, who then met in pairs of two, followed by all four reaching consensus on identified themes. The findings revealed six major themes: enriching the IGL experiences; passion for teaching and interacting with young people; transition to and life after retirement; impact of COVID on engagement; university role in connecting with and engaging emeriti; and satisfaction with values and ideas expressed in IGL activities. These themes offer guidance in promoting AFU principle 9 through a novel intergenerational activity that benefits emeriti professors, students and universities.

